# Recovery rate and determinants of severe acute malnutrition children treatment in Ethiopia: a systematic review and meta-analysis

**DOI:** 10.1186/s13643-019-1249-4

**Published:** 2019-12-13

**Authors:** Kassahun Gebeyehu Yazew, Chanyalew Worku Kassahun, Amare Wondim Ewnetie, Habtamu Kerebih Mekonen, Endalamaw Salilew Abagez

**Affiliations:** 1 School of Nursing, College of Medicine and Health Science, University of Gondar, Gondar, Ethiopia; 2 Department of Psychiatric, College of Medicine and Health Science, University of Gondar, Gondar, Ethiopia

**Keywords:** Recovery rate, Therapeutic feeding unit, Severe acute malnutrition, Under-five children, Children, Ethiopia

## Abstract

**Background:**

Severe acute malnutrition affects more than 20 million children. Africa is pointed out as a region where the problem is highly prevalent. There were individual studies on the recovery rate and its determinants among children with severe acute malnutrition in Ethiopia. But, there is no national pooled estimate. Therefore, this systematic review and meta-analysis aimed to estimate the recovery rate and determinants among children with severe acute malnutrition admitted to the therapeutic feeding unit in Ethiopia.

**Methods:**

The Preferred Reporting Items for Systematic Reviews and Meta-Analyses guideline was followed in this study. Studies were accessed through electronic web-based search from PubMed, Cochrane Library, Google Scholar, and EMBASE. The statistical analysis was conducted using STATA version-11 software. The pooled prevalence was estimated with 95% confidence intervals using a random-effects model.

**Result:**

A total of 12 studies were included with 2658 participants in the analysis. The overall pooled estimated recovery rate among children with severe acute malnutrition admitted to the inpatient therapeutic feeding unit in Ethiopia was 72.02 % (CI, 64.83, 79.22%). In the subgroup analysis, the highest estimate (80.29%) was observed in studies conducted in Oromia regional state, while 68.63% was observed in studies Southern Nation Nationality of people region 68.63%. Children who had no congestive heart failure were 4.88 times (OR, 4.88; 95% CI, 2.246, 10.586) more likely to recover than their counterparts.

**Conclusion:**

The recovery rate among severe acute malnourished children on the therapeutic feeding unit in Ethiopia lied within the international minimum sphere. Hence, health care providers shall strengthen the management of severe acute malnutrition and management other co-morbidities like congestive heart failure.

**Systematic review registration:**

PROSPERO CRD42019119124

## Background

Severe acute malnutrition (SAM) is stated as a weight-for-height measurement of 70% or more below the median, or three SD or more below the mean National Center for Health Statistics reference points, which is called “wasted”; the occurrence of two-sided pitting edema of nutritional origin, which is called “oedematous malnutrition”; or a mid-upper-arm circumference of less than 110 mm in children aged 1–5 years [1].

Globally, it is valued that there are nearly 20 million children who are severely acutely malnourished. Most of them live in South Asia and Sub-Saharan Africa [2].

Malnutrition can affect all age groups but is more frequent among infants and young children (4–6 years). Malnutrition contributes to 50–60% of the child deaths for which infection is the underlying cause [3, 4]. A literature review conducted in Africa showed that children with SAM given RUTF were 51% more likely to achieve nutritional recovery than the standard care group [5].

In developing countries, 2% of children suffer from severe acute malnutrition [6]. In India, 2.8% of children under five are severely wasted [7]. The United Nations Children’s Fund estimated sixty thousand children to be severely malnourished in Ethiopia [8]. In children younger than 5 years of age, according to the Ethiopian Demographic and Health Survey (EDHS) report, 11% are wasted, 2% severely wasted, 38% underweight (below – 2 SD), and 16% severely underweight (below – 3 SD) [9]. Among the principal causes of death in young children, 60.7% of deaths from diarrhea, 52.3% of deaths from pneumonia, 44.8% of deaths from measles, and 57.3% of deaths from malaria are attributable to under-nutrition [10]. According to the United Nations International Emergency Fund (UNICEF) estimates, around 26 million under-five children suffer from SAM in developing countries [11].

Despite the huge effects of the recovery rate in the treatment of children with severe acute malnutrition and its importance as a public health problem in Ethiopia, the overall recovery rate among children treated with severe acute malnutrition in the country level remains unknown. Therefore, the objective of this systematic review and meta-analysis is to estimate the pooled recovery rate and its determinants among children with severe acute malnutrition admitted to the inpatient therapeutic feeding unit in Ethiopia.

## Materials and methods

### Study protocol registration

PROSPERO database with protocol number CRD42019119124.

### Search strategy

We made an inclusive literature search conducted from October 2018 to January 2018 from PubMed, Cochrane Library, Google Scholar, CINAHL, and EMBASE. A selection of publications, data extraction, and reported results for the review was designed in accordance with the Preferred Reporting Items for Systematic Reviews and Meta-Analyses (PRISMA) guidelines [12]. Throughout the comprehensive literature search, the following search terms were used: “Recovery Rate and Determinants of the Treated Severe Acute Malnutrition Children in Ethiopia,” “Recovery rate OR treatment outcome in Ethiopia,” and “Recovery rate AND children in Ethiopia.” Furthermore, we checked the reference lists of published studies to identify additional articles.

### Selection and identification of studies

**Fig. 1:**
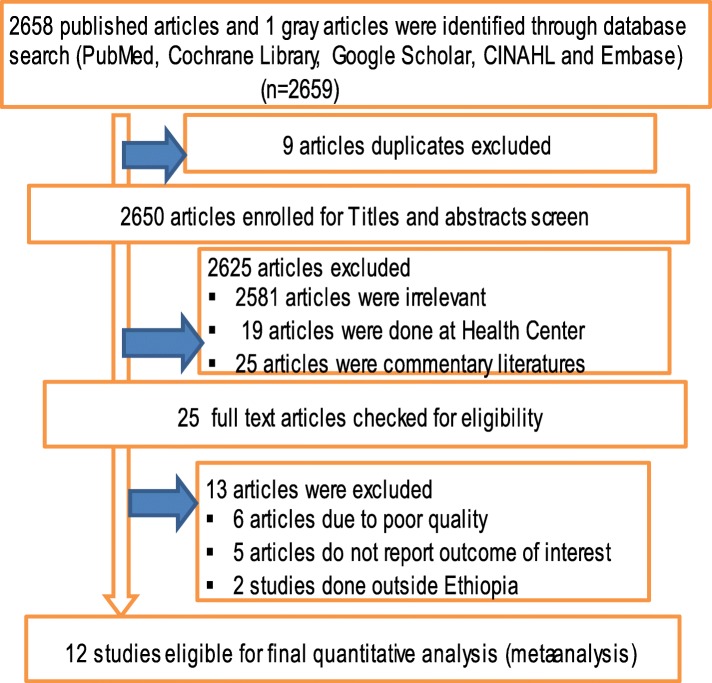
Flow chart of study selection for meta-analysis of recovery rate of the treated severe acute malnutrition children in Ethiopia, 2018

A total of 2658 studies were identified from the literature search. We added one gray literature that was not found in the search. Of these studies, 9 articles of duplicate records were identified and removed. A total of 2625 articles were excluded after reviewing the titles and abstracts (because 2581articles were irrelevant and 19 articles were done at Health Center). After assessing the full texts of the remaining articles, 13 additional articles were excluded because of the following: 6 articles are of poor quality, 5 articles do not report outcome interest, and 2 articles were done outside Ethiopia. Therefore, a total of twelve unique studies were eligible and enrolled for final analysis (Fig. 1).

### Study selection and eligibility criteria

We included all studies that were conducted on the recovery rate and determinants of severe acute malnutrition children treatment. The participants were SAM children with age between 0 and180 months, regardless of their sex and other characteristics. We included all article types that were published in the form of journal articles, master’s thesis, and dissertations in English. Moreover, studies which failed to report recovery rate and those studies conducted on adult were excluded. No restriction was made to the date of publication that was conducted only in Ethiopia.

### Outcome measures

#### Research questions

1. What is the estimated pooled prevalence of the recovery rate among children with severe acute malnutrition admitted to the inpatient therapeutic feeding unit?
2. What are the determinants that affect the recovery rate among children with severe acute malnutrition admitted to the inpatient therapeutic feeding unit?

The primary outcome of this systematic review and meta-analysis was measuring the prevalence of the recovery rate among children with severe acute malnutrition admitted to the inpatient therapeutic feeding unit presented as a percentage of the overall participants.

The secondary outcome was assessed determinants influencing the recovery rate among children with severe acute malnutrition admitted to the inpatient therapeutic feeding unit.

### Quality assessment and critical appraisal

The qualities of each article were assessed by using a critical appraisal tool (JBI) for use in systematic reviews of the prevalence study [13]. Those studies less than 50% of the quality assessment checklist criteria were excluded. The methodological quality of the identified articles was also assessed by two reviewers (K.G. and C.W.) independently, and disagreements among reviewers were fixed accordingly through a discussion with a third reviewer (A.W) when necessary

### Data extraction

Data were extracted using pre-piloted data extraction forms which were developed by the two authors (E.S. and H.K). It included names of author, year of publication, interventions, durations, the region in the country, study design, sample size, number of people with the outcome, and overall prevalence.

### Data analysis and synthesis

The extracted data in a Microsoft Excel spreadsheet were imported to STATA v. 11 for analysis. The analysis was done by the authors using STATA. A random-effects model was used to estimate the overall pooled magnitude. The DerSimonian and Laird method, which assumes heterogeneity across studies, is the most common method for using a random-effects model in the meta-analysis [14, 15]. A random-effects meta-analysis is also recommended for use when heterogeneity between studies exists [16]. The heterogeneity of articles was tested using 퐼^2^ test statistics, that ranged from 0 to 100%. A value of 0% indicates no observed heterogeneity, while 100% indicates significant heterogeneity. A *p* value of less than 0.05 was used to declare heterogeneity [17]. In this meta-analysis, *I*^2^ values were found to be high (> 75%). Moreover, the presence of heterogeneity was also assessed by subgroup analysis and meta-regression. A visual inspection of the publication bias was conducted using a funnel plot. An asymmetry of the funnel plot is an indicator of publication bias [18]. Eggers and Begg’s tests were also conducted to check the potential publication bias. A *p* value of less than 0.05 was used to declare the statistical significance of publication bias [19]. Additionally, the sensitivity analysis was also done to assess whether the pooled prevalence estimates were affected by single studies.

## Results

### Characteristics of included studies

A total of 12 studies with 4890 participants were included in this meta-analysis and are summarized in Table 1. The studies were conducted from 2008 to 2018 in different regions of the country. Among 12 studies, five of them [22, 26–28, 30] were in the Amhara region, two studies [24, 31] were in SNNP, four studies [20, 21, 23, 25] were in the Oromia region, and one study [29] was from the Dredewa region. A study in both minimum [25] and maximum [20] sample size was conducted in the Oromia region. In terms of study design, 3 studies [21, 22, 28] were conducted by cross-sectional, 7 studies [22, 23, 25–298, 31] were conducted by retrospective cohort, and two studies [23, 25] were conducted by retrospective case-control (Table 1).

**Table 1:** Characteristics of studies included in meta-analysis of SAM recovery rate in Ethiopia, 2018

S. no	Author/s (reference)	Publication year	Interventions	Durations in days	Age in months	Region	Study design	Sample size	Case	Prevalence% (95% CI)
1	Jarso et al. [20]	2015	TF	17.4	6–59	Ormia	Cohort	947	737	77.8 (75.15,80.45)
2	MB Mena et al. [21]	2018	TF + M	21	0–180	Ormia	CS	205	137	66.8 (60.35,73.25)
3	Abeje AT, et al. [22]	2016	TF	35	2–59	Amhr	CS	298	204	68.5 (63.23,73.77)
4	A. Berti et al. [23]	2008	TF	12.5	0–59	Orm	CC	493	436	88.4 (85.57,91.23)
5	Kabeta A et al. [24]	2017	TF + M	18.16	0–59	SNNP	Cohort	196	153	78 (72.20, 83.80)
6	Chalachew M et al. [25]	2014	TF	21	0–168	Ormia	CC	173	150	87 (74.34, 83.26)
7	Mekuria et al. [26]	2017	TF + M	11	6–59	Amhra	Cohort	253	197	77.9 (72.79, 83.01)
8	T Chane et al. [27]	2014	TF + M	14	0–59	Amhara	Cohort	324	275	85 (81.11, 88.89)
9	Desyibelew HD et al. [28]	2017	TF + M	23	6–59	Amhara	CS	401	234	58.4 (53.58, 63.22)
10	Oumer et al. [29]	2016	TF + M	9	0–59	Diriedewa	Cohort	617	431	69.9 (66.28, 73.52)
11	Desta et al. [30]	2015	TF	32	0–59	Amhara	Cohort	415	193	46.5 (41.70,51.30)
12	Tadele Girum et al. [31]	2017	TF + M	14	0–59	SNNP	Cohort	568	338	59.5 (55.46,63.54)

CC case-control, CS cross-sectional, M medications, TF therapeutic foods, SNNP Southern Nation and Nationalities of people

Therapeutic foods include F75, F100 and plump net

Medications are drugs given during admission with therapeutic foods (Amoxicillin, Ampicillin, Gentamycin, Vitamin A, Folic acid, Albendazole/Mebendazole)

### The recovery rate of the treatment of SAM children (meta-analysis)

**Fig. 2:**
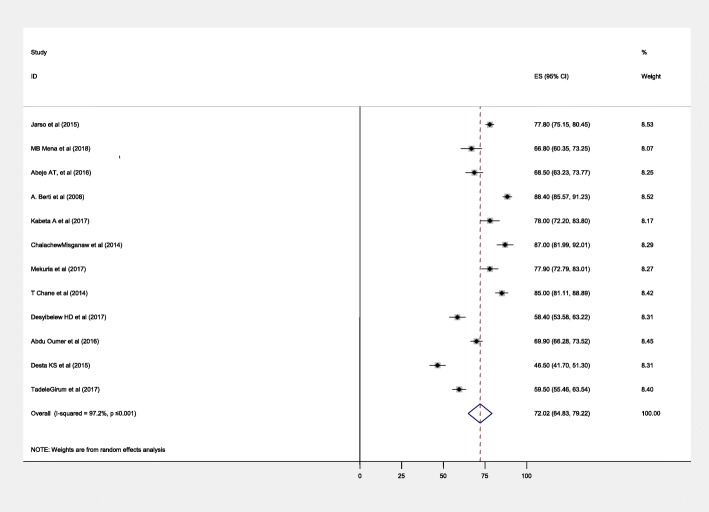
Forest plot showing the pooled recovery rate of the treated SAM children in Ethiopia, 2018

The estimated pooled recovery rate of the treatment of SAM children reported by the 12 studies was 72.02 (95% CI, 64.83, 79.22%) with significant heterogeneity between studies (*I*^2^ = 97.2%, *p* ≤ 0.001) (Fig. 2).

In the subgroup analysis by the study area and study designs, the highest estimated recovery rates of 80.29%, 95% CI 72.27, 88.32, *I*^2^ = 94.5% and 68.63%, 95% CI 50.50, 86.76%, *I*^2^ = 96.2% were found in studies conducted in Oromia and SNNP regional states respectively (Additional file 1: Figure S1). Case-control studies account the highest estimate (88.06%, 95% CI 85.6, 90.52, *I*^2^ = 0.0%) (Additional file 2: Figure S2).

### Investigation of heterogeneity

Heterogeneity in systematic reviews and meta-analysis results of studies are inevitable due to the difference in study quality, methodology, sample size, and inclusion criteria for participants [32, 33]. In this meta-analysis, the value of *I*^2^ is definitely an indication of significantly high heterogeneity, so we conducted the analysis with a random-effects model to adjust for the observed variability. Furthermore, the presence of heterogeneity was also assessed by subgroup analysis (study area and study designs). However, the level of heterogeneity was high after the subgroup analysis discussed above (Additional file 1: Figure S1 and Additional file 2: Figure S2). Then, we further try to investigate the source of heterogeneity using a meta-regression model with publication year and sample size as covariates. Meta-regression is a more complex and preferable method than subgroup analysis for investigating heterogeneity and has the potential advantage of allowing the assessment of one or more covariates simultaneously [34]. The result of the meta-regression analysis showed that both covariates were not statistically significant for the presence of the heterogeneity (Additional file 3: Table S1).

### Publication bias

The presence of publication bias was examined using funnel plots and tests (Egger’s and begs). Each point in funnel plots represents no separate study, and symmetrical distribution is evidence of the absence of publication bias [35]. First, each study’s effect size was not plotted against the standard error and visual inspection of the funnel plot suggests asymmetry, as 3 studies lay on the right side and nine studies on midline representing the pooled prevalence (Additional file 4: Figure S3). We also performed Egger’s, and Bag’s tests to investigate the publication bias. The result of these tests was not showing the significant evidence of the publication bias (*p* value > 0.05) (Additional file 5: Table S2).

### Sensitivity analysis

The result indicated that no single study unduly influenced the overall estimate of the recovery rate among children with SAM on treatment (Additional file 6: Figure S4).

### Factors assessed

Seven studies [21, 22, 24, 27–30] were included in the analysis of determinant factors of the recovery rate. Greater heterogeneity was observed among studies. Five factors were assessed in the quantitative meta-analysis. Six articles for the presence of HIV/AIDS [21, 22, 27–30] with 2828 participants, two articles for the presence of CHF [27, 28] with 725 participants, three articles for the presence of anemia [21, 24, 28] with 802 participants, and five articles for presence of TB [24, 27–30] with 1953 participants were assessed for their associations to the recovery rate of treated SAM children.

Of the factors, the presence of CHF was the significant factors of the recovery rate. Children who had no congestive heart failure were 4.88 times (OR 4.88, 95% CI 2.246, 10.586) more likely to recover than their counterparts (Additional file 7: Figure S5).

## Discussion

Despite the effort implemented to reduce malnutrition, the proportion of severe acute malnutrition treatment recovery rate in the therapeutic feeding unit is still lower. As far as is known, there are no previous systematic reviews/meta-analyses that have examined the national estimate of the recovery rate and its determinants among severe acute malnutrition among children in Ethiopia.

The result of this meta-analysis indicated that the overall recovery rate among admitted children with SAM to therapeutic feeding unit was 72.02. This finding is in line with the international standard recommendation of the minimum recovery rate is greater than 75% [36]. It is also similar to findings conducted in 13 African countries 73% [37] and Kenya 73.3% [38] studies. This finding is higher than the studies done from different countries; in India 51.7% [39], Pakistan 50% [40], and in low and middle-income countries 51% [5]. The difference might be due to differences in study design, study population, socioeconomic status, quality of care provided for children, health-seeking behavior, availability, and the accessibility of therapeutic foods and medications. However, the result of this finding was lower than studies recruited at Bangladesh 92% [41], Niger 91.4% [42], Malawi 89% [43], and Sudan 82% [44]. The possible justification can be sample size and study setting.

The results of this review highlighted common determinant factors for the recovery rate among children with SAM on the treatment. Even though there is significant heterogeneity in odds ratios, the study had an odds ratio of greater than one which indicates an increased risk for recovery rate among children with SAM on treatment.

As it is indicated in the seven studies, the presence of CHF was the significant determinant factors of recovery rate among children with SAM on treatment in Ethiopia. The outcome of this finding was supported by the studies conducted in low- and middle-income countries [5] and South Africa [45].

## Conclusion

The proportion of the recovery rate lied on the minimum sphere of the international standard for the treated SAM children. The present of CHF affects the recovery rate of severe acute malnutrition treatment among children in Ethiopia. Hence, health care providers shall strengthen the management of severe acute malnutrition and management other co-morbidities like CHF.

## Potential limitations

Like other systematic review and meta-analysis, this review has some drawbacks. The first drawback of this review was only English articles, or reports were considered to conduct this nationally based review. In addition, some of the studies included in this review were cross-sectional in nature because the outcome variable might be affected by other confounding variables. Hence, this factor could affect the estimated result. Furthermore, this review represented only studies reported from four regions of the country. Therefore, the regions may be under-represented due to the limited number of articles included.

## Supplementary information

**Additional file 1: Figure S1.** Subgroup analysis by regions on the recovery rate of the treatment among SAM children Ethiopia, 2018

**Additional file 2: Figure S3.** Subgroup analysis by study designs on treatment recovery rate among SAM children Ethiopia, 2018

**Additional file 3: Table S1.** Meta-regression analysis of factors with heterogeneity of the recovery rate of children with SAM treatment in Ethiopia, 2018

**Additional file 4: Figure S2.** Funnel plots to test, the publication bias of the 12 studies, 2018

**Additional file 5: Table S2.** Publication bias of recovery rate of the treatment among SAM children in Ethiopia, 2018

**Additional file 6: Figure S3.** Result of Sensitivity analysis of the 12 studies, 2018

**Additional file 7: Figure S4.** Forest plot depicting the pooled odds ratio (log scale) of the associations between recovery rate and its determinant (Presence of CHF), 2018

**Additional file 8.** PRISMA checklist

## Data Availability

Data will be available on the request of the corresponding author.

## Abbreviations

CHF: Chronic heart failure
DD: Driedewa
HIV/AIDS: Human immune virus/acquired immune deficiency syndrome
JBI: Joanna Brigg’s Institute
OR: Odds ratio
SAM: Severe acute malnutrition
SNNP: Southern Nation, Nationality of people
TFP: Therapeutic feeding program
