# Supplementary material for: Recovery rate and determinants of severe acute malnutrition children treatment in Ethiopia: a systematic review and meta-analysis
Source: Syst Rev. 2019 Dec 13;8:323. doi: 10.1186/s13643-019-1249-4 (PMC6911294; doi:10.1186/s13643-019-1249-4)
Supplement: Supplementary file 1 — Additional file 1: Figure S1. Subgroup analysis by regions on the recovery rate of the treatment among SAM children Ethiopia, 2018 [file 13643_2019_1249_MOESM1_ESM.docx]

**Additional file1**

NOTE: Weights are from random effects analysis

.

.

.

.

Overall (I-squared = 97.2%, p ≤0.001)

Subtotal (I-squared = 97.8%, p ≤0.001)

Jarso et al

A. Berti et al

Desta KS et al

Author

Abdu Oumer et al

TadeleGirum et al

Kabeta A et al

Mekuria et al

T Chane et al

DD

ChalachewMisganaw et al

Subtotal (I-squared = .%, p ≤0 .001)

Subtotal (I-squared = 96.2%, p ≤0.001)

Amhr

Orm

Subtotal (I-squared = 94.5%, p ≤ 0.001)

Desyibelew HD et al

SNNP

MB Mena et al

Abeje AT, et al

2015

2008

2015

year

2016

2017

2017

2017

2014

2014

2017

2018

2016

72.02 (64.83, 79.22)

67.28 (53.10, 81.46)

77.80 (75.15, 80.45)

88.40 (85.57, 91.23)

46.50 (41.70, 51.30)

ES (95% CI)

69.90 (66.28, 73.52)

59.50 (55.46, 63.54)

78.00 (72.20, 83.80)

77.90 (72.79, 83.01)

85.00 (81.11, 88.89)

87.00 (81.99, 92.01)

69.90 (66.28, 73.52)

68.63 (50.50, 86.76)

80.29 (72.27, 88.32)

58.40 (53.58, 63.22)

66.80 (60.35, 73.25)

68.50 (63.23, 73.77)

100.00

41.56

8.53

8.52

8.31

Weight

8.45

8.40

8.17

8.27

8.42

%

8.29

8.45

16.58

33.41

8.31

8.07

8.25

72.02 (64.83, 79.22)

67.28 (53.10, 81.46)

77.80 (75.15, 80.45)

88.40 (85.57, 91.23)

46.50 (41.70, 51.30)

ES (95% CI)

69.90 (66.28, 73.52)

59.50 (55.46, 63.54)

78.00 (72.20, 83.80)

77.90 (72.79, 83.01)

85.00 (81.11, 88.89)

87.00 (81.99, 92.01)

69.90 (66.28, 73.52)

68.63 (50.50, 86.76)

80.29 (72.27, 88.32)

58.40 (53.58, 63.22)

66.80 (60.35, 73.25)

68.50 (63.23, 73.77)

100.00

41.56

8.53

8.52

8.31

Weight

8.45

8.40

8.17

8.27

8.42

%

8.29

8.45

16.58

33.41

8.31

8.07

8.25

0

.1

1

10

Figure s1: Subgroup analysis by regions on treatment recovery rate among SAM children Ethiopia, 2018
