# Supplementary material for: Recovery rate and determinants of severe acute malnutrition children treatment in Ethiopia: a systematic review and meta-analysis
Source: Syst Rev. 2019 Dec 13;8:323. doi: 10.1186/s13643-019-1249-4 (PMC6911294; doi:10.1186/s13643-019-1249-4)
Supplement: Supplementary file 2 — Additional file 2: Figure S3. Subgroup analysis by study designs on treatment recovery rate among SAM children Ethiopia, 2018 [file 13643_2019_1249_MOESM2_ESM.docx]

**Additional file 2**


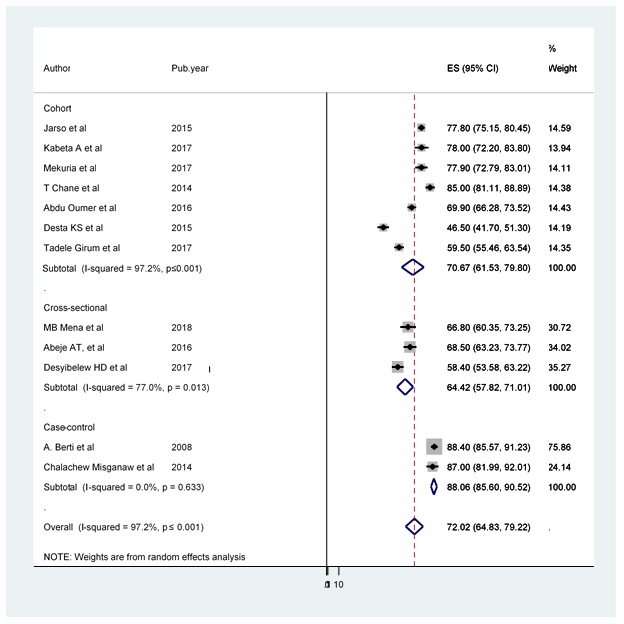
Figure s3: Subgroup analysis by study designs on treatment recovery rate among SAM children Ethiopia, 2018
