# Supplementary material for: Recovery rate and determinants of severe acute malnutrition children treatment in Ethiopia: a systematic review and meta-analysis
Source: Syst Rev. 2019 Dec 13;8:323. doi: 10.1186/s13643-019-1249-4 (PMC6911294; doi:10.1186/s13643-019-1249-4)
Supplement: Supplementary file 3 — Additional file 3: Table S1. Meta-regression analysis of factors with heterogeneity of the recovery rate of children with SAM treatment in Ethiopia, 2018 [file 13643_2019_1249_MOESM3_ESM.docx]

Additional file 3

Table s1: Meta-regression analysis of factors with heterogeneity of treatment recovery rate in Ethiopia, 2018

| **Heterogeneity source** | **Coefficients** | **Std. err.** | **t** | **P>t** | **[95% Conf. Interval]** |
| --- | --- | --- | --- | --- | --- |
|  |  |  |  |  |  |
| Publication year | -.032803 | .1933166 | -0.17 | 0.869 | -.4701154, .4045095 |
| Sample size | 8.41e-06 | .0023964 | 0.00 | 0.997 | -.0054126, .0054294 |
| _cons | 70.3707 | 389.4571 | 0.18 | 0.861 | -810.6426, 951.384 |
