# Supplementary material for: Recovery rate and determinants of severe acute malnutrition children treatment in Ethiopia: a systematic review and meta-analysis
Source: Syst Rev. 2019 Dec 13;8:323. doi: 10.1186/s13643-019-1249-4 (PMC6911294; doi:10.1186/s13643-019-1249-4)
Supplement: Supplementary file 4 — Additional file 4: Figure S2. Funnel plots to test, the publication bias of the 12 studies, 2018 [file 13643_2019_1249_MOESM4_ESM.docx]

Additional file 4

Figure s3: Funnel plots to test the publication bias of the 12 studies, 2018
