# Supplementary material for: Recovery rate and determinants of severe acute malnutrition children treatment in Ethiopia: a systematic review and meta-analysis
Source: Syst Rev. 2019 Dec 13;8:323. doi: 10.1186/s13643-019-1249-4 (PMC6911294; doi:10.1186/s13643-019-1249-4)
Supplement: Supplementary file 5 — Additional file 5: Table S2. Publication bias of recovery rate of the treatment among SAM children in Ethiopia, 2018 [file 13643_2019_1249_MOESM5_ESM.docx]

**Additional file5**

Table s2: Publication bias oftreatmentrecovery rateamong SAM children in Ethiopia, 2018

| Std_Eff | Coefficients | Std. err. | t | P>t | [95% Conf. Interval] |
| --- | --- | --- | --- | --- | --- |
| slope  bias | 4.556975 | .1850576 | 24.62 | 0.001 | 4.14464, 4.969309 |
|  | -.129971 | .0892108 | -1.46 | 0.176 | -.3287451, .0688031 |
