# Supplementary material for: Recovery rate and determinants of severe acute malnutrition children treatment in Ethiopia: a systematic review and meta-analysis
Source: Syst Rev. 2019 Dec 13;8:323. doi: 10.1186/s13643-019-1249-4 (PMC6911294; doi:10.1186/s13643-019-1249-4)
Supplement: Supplementary file 6 — Additional file 6: Figure S3. Result of Sensitivity analysis of the 12 studies, 2018 [file 13643_2019_1249_MOESM6_ESM.docx]

**Additional file 6**

63.14

72.02

64.83

79.22

80.64

Jarso et al (2015)

MB Mena et al (2018)

Abeje AT, et al (2016)

A. Berti et al (2008)

Kabeta A et al (2017)

ChalachewMisganaw et al (2014)

Mekuria et al (2017)

T Chane et al (2014)

Desyibelew HD et al (2017)

Abdu Oumer et al (2016)

Desta KS et al (2015)

TadeleGirum et al (2017)

Lower CI Limit

Estimate

Upper CI Limit

Meta-analysis estimates, given named study is omitted

Figure s4: Result of Sensitivity analysis of the 12 studies, 2018
