# Supplementary material for: Recovery rate and determinants of severe acute malnutrition children treatment in Ethiopia: a systematic review and meta-analysis
Source: Syst Rev. 2019 Dec 13;8:323. doi: 10.1186/s13643-019-1249-4 (PMC6911294; doi:10.1186/s13643-019-1249-4)
Supplement: Supplementary file 7 — Additional file 7: Figure S4. Forest plot depicting the pooled odds ratio (log scale) of the associations between recovery rate and its determinant (Presence of CHF), 2018 [file 13643_2019_1249_MOESM7_ESM.docx]

**Additional file 7**

Figure s5: Forest plot depicting pooled odds ratio of the associations with Recovery Rate, 2018
