# Supplementary material for: Recovery rate and determinants of severe acute malnutrition children treatment in Ethiopia: a systematic review and meta-analysis
Source: Syst Rev. 2019 Dec 13;8:323. doi: 10.1186/s13643-019-1249-4 (PMC6911294; doi:10.1186/s13643-019-1249-4)
Supplement: Supplementary file 8 — Additional file 8. PRISMA checklist [file 13643_2019_1249_MOESM8_ESM.docx]

**Additional file 8: PRISMA checklist**

| **Section/topic** | | **#** | | | **Checklist item** | | | **Reported on page #** |
| --- | --- | --- | --- | --- | --- | --- | --- | --- |
| **TITLE** | | | | | | | |  |
| Title | | **1** | | | Recovery Rate and Determinants of Severe Acute Malnutrition Children Treatment in Ethiopia: A systematic review and Meta-Analysis | | | **1** |
| **ABSTRACT** | | | | | | | |  |
| Structured summary | | **2** | | | **Background:** Severe acute malnutrition affects more than 20 million children. Africa is pointed out as a region where the problem is highly prevalent. There were individual studies on the recovery rate and its determinants among children with severe acute malnutrition in Ethiopia. But, there is no national pooled estimate. Therefore, this systematic review and meta-analysis aimed to estimate the recovery rate and determinants among Children with Severe Acute Malnutrition Admitted to the therapeutic feeding unit in Ethiopia.  **Methods:** The Preferred Reporting Items for Systematic Reviews and Meta-Analyses guideline was followed in this study. Studies were accessed through electronic web-based search from PubMed, Cochrane Library, Google Scholar, and EMBASE. The statistical analysis was conducted using STATA version-11 software. The pooled prevalence was estimated with 95% confidence intervals using a random-effects model.  **Result:** A total of 12 studies were included with 2, 658 participants in the analysis. The overall pooled estimated recovery rate among Children with Severe Acute Malnutrition Admitted to the inpatient therapeutic feeding unit in Ethiopia was 72.02 % (CI: 64.83, 79.22 %). In the sub-group analysis, the highest estimate (80.29%) was observed in studies conducted in Oromia regional state while 68.63% was observed in studies Southern Nation Nationality of people region 68.63%. Children who had no congestive heart failure were 4.88 times (OR: 4.88, 95% CI: 2.246, 10.586) more like to recover than their counterparts.  ***Conclusion*:** The recovery rate among severe acute malnourished children on the therapeutic feeding unit in Ethiopia lied within the international minimum sphere. Hence, health care providers shall strengthen the management of severe acute malnutrition and management other co morbidities like congestive heart failure.  **Study protocol registration**: It is registered data base: (CRD42019119124).  Key: - Recovery rate, therapeutic feeding unit, severe acute malnutrition, under-five children, children, Ethiopia | | | **2** |
| **INTRODUCTION** | | | | | | | |  |
| Rationale | **3** | | | | The aim of this systematic review and meta-analysis is to estimate the pooled recovery rate and its determinants among Children with Severe Acute Malnutrition Admitted to the Inpatient Therapeutic Feeding unit in Ethiopia | | | **3** |
| Objectives | **4** | | | | **1**/What is the estimated pooled prevalence of recovery rate among children with severe acute malnutrition admitted to the inpatient therapeutic feeding unit?**2**/What are the determinants that affect the recovery rate among children with severe acute malnutrition admitted to the inpatient therapeutic feeding unit? | | | **3 and 5** |
| **METHODS** | | | | | | | |  |
| Protocol and registration | | | **5** | | | It is registered data base at PROSPERO: (CRD42019119124) | | **4** |
| Eligibility criteria | | | **6** | | | We included all studies that were conducted on the recovery rate and determinants of severe acute malnutrition Children treatment. The participants were SAM children with age between 0 to180^th^ months, regardless of their sex and other characteristics. We included all article types that were published in the form of journal articles, master’s thesis, and dissertations in English. Moreover, pieces of literature which failed to report recovery rate and those studies conducted on adult were excluded. No restriction was made to date of publication than conducted only in Ethiopia. | | **4** |
| Information sources | | | **7** | | | We made an inclusive literature search from PubMed, Cochrane Library, Google Scholar, CINAHL and EMBASE were conducted from October/2018 to January/ 2018 | | **4** |
| Study selection | | | **9** | | | A total of 2658 studies were identified from the literature search. We added one gray literature that was not found in the search. | | **4** |
| Data collection process | | | **10** | | | Data were extracted using pre-piloted data extraction forms which were developed by the two authors (E.S. and H.K). | | **5** |
| Data items | | | **11** | | | The primary outcome of this systematic review and Meta-analysis was measuring the prevalence of recovery rate among children with severe acute malnutrition admitted to the inpatient therapeutic feeding unit presented as a percentage of the overall participants. The secondary outcome was assessed determinants influencing the recovery rate | | **5** |
| Risk of bias in individual studies | | | **12** | | | The extracted data in a Microsoft Excel spreadsheet were imported to STATA v. 11 for analysis. The analysis was done by the authors using STATA | | **5** |
| Summary measures | | | **13** | | | Table 1: Characteristics of studies included in meta-analysis of SAM recovery rate in Ethiopia | | **6and Table 1** |
| Synthesis of results | | | **14** | | | The heterogeneity of articles was tested using 𝐼^2^ test statistics, ranges from 0 to 100%. | | **5 and 6** |
| Risk of bias across studies | | | **15** | | | Eggers and Begg’s tests were also conducted to check the potential publication bias. | | **6** |
| Additional analyses | | | **16** | | | the sensitivity analysis was also done to assess whether the pooled prevalence estimates were affected by single studies | | **6** |
| RESULTS | | | | | | | |  |
| Study selection | | | | **17** | | | total of 12 studies with 4890 participants were included in this meta-analysis are summarized in Table1 | **6** |
| Study characteristics | | | | **18** | | | Among 12 studies five of them ([20-24](#_ENREF_20)) were in Amhara region, two studies ([25](#_ENREF_25), [26](#_ENREF_26)) were in SNNP, four studies ([27-30](#_ENREF_27)) were in Oromia region and one([31](#_ENREF_31)) was from Dredewa region. The study both minimum ([30](#_ENREF_30)) and the maximum ([28](#_ENREF_28)) sample size was conducted in Oromia region. In terms of study design 3 studies ([21](#_ENREF_21), [24](#_ENREF_24), [29](#_ENREF_29)) were conducted by cross-sectional, 7 studies ([22](#_ENREF_22), [23](#_ENREF_23), [25-28](#_ENREF_25), [31](#_ENREF_31)) were conducted by retrospective cohort and two studies ([27](#_ENREF_27), [30](#_ENREF_30)) were conducted by retrospective case-control (Table1). | **6** |
| Risk of bias within studies | | | | **19** | | | First, each study’s effect size was not plotted against the standard error and visual inspection of the funnel plot suggests asymmetry, as 3 studies lay on the right side and nine studies on midline representing the pooled prevalence (Additional file 4: Figure s3). We also performed Egger’s, and Bag’s tests to investigate the publication bias. The result of these tests was not showing the significant evidence of the publication bias (p value>0.05) (Additional file 5: table s2). | **7; Additional file 4 and 5** |
| Results of individual studies | | | | **20** | | | The estimated pooled recovery rate of the treatment of SAM children reported by the 12 studies was 72.02 (95% CI: 64.83, 79.22 %) with significant heterogeneity between studies (I^2^= 97.2%, p ≤ 0.001) (Figure 2). | **6** |
| Synthesis of results | | | | **21** | | | Seven studies were included in the analysis of determinant factors of the recovery rat. | **7-8** |
| Risk of bias across studies | | | | **22** | | | Presence of publication bias was examined using funnel plots and tests (Egger’s and begs). | **6** |
| Additional analysis | | | | **23** | | | In the subgroup analysis by study area and study designs, the highest estimated recovery rate (80.29 %, 95% CI: 72.27, 88.32, I^2^= 94.5%) and (68.63%, 95% CI: 50.50, 86.76%, I^2^= 96.2%) were found in studies conducted in Oromia and SNNP regional states respectively (Additional file1: Figure s1), Case-control studies account the highest estimate (88.06%, 95% CI: 85.6, 90.52, I^2^= 0.0%) (Additional file 2: Figure s2).Sensitivity Analysis: - The result indicated, no single study unduly influenced the overall estimate of recovery rate among children with SAM on treatment | **6 and 7; Additional file 1 and 2** |
| **DISCUSSION** | | | | | | | |  |
| Summary of evidence | **24** | | | | Despite the effort implemented to reduce malnutrition, the proportion of severe acute malnutrition treatment recovery rate in the therapeutic feeding unit is still lower. | | | **8** |
| Limitations | **25** | | | | Like other systematic review and meta-analysis, this review has some drawbacks. The first drawback of thisreview was only English articles or reports were considered to conduct this nationally based review. In addition, some of the studies included in this review were cross-sectional in nature duet that; the outcome variable might be affected by other confounding variables. Hence, this factor could affect the estimated result. Furthermore, this review represented only studies reported from four regions of the country. Therefore, the regions may be under-represented due to the limited number of articles included. | | | **9** |
| Conclusions | **26** | | | | The proportion of the recovery rate lied on the minimum sphere of the international standard for the treated SAM children. | | | **9** |
| **FUNDING** | | | | | | | |  |
| Funding | **27** | | | | Not applicable | | | **10** |
